# Characterization of a Putative Spindle Assembly Checkpoint Kinase Mps1, Suggests Its Involvement in Cell Division, Morphogenesis and Oxidative Stress Tolerance in *Candida albicans*

**DOI:** 10.1371/journal.pone.0101517

**Published:** 2014-07-15

**Authors:** Mohan Kamthan, Vijaya Kumar Nalla, Deepa Ruhela, Ayushi Kamthan, Protiti Maiti, Asis Datta

**Affiliations:** 1 National Institute of Plant Genome Research, New Delhi, India; 2 Department of Microbiology, Bhaskaracharya college, University of Delhi, New Delhi, India; Florida State University, United States of America

## Abstract

In *Saccharomyces cerevisiae MPS1* is one of the major protein kinase that governs the spindle checkpoint pathway. The *S. cerevisiae* structural homolog of opportunistic pathogen *Candida albicans CaMPS1*, is indispensable for the cell viability. The essentiality of Mps1 was confirmed by Homozygote Trisome test. To determine its biological function in this pathogen conditional mutant was generated through regulatable *MET3* promoter. Examination of heterozygous and conditional (+Met/Cys) *mps1* mutants revealed a mitosis specific arrest phenotype, where mutants showed large buds with undivided nuclei. Flowcytometry analysis revealed abnormal ploidy levels in *mps1*mutant. In presence of anti-microtubule drug Nocodazole, *mps1* mutant showed a dramatic loss of viability suggesting a role of Mps1 in Spindle Assembly Checkpoint (SAC) activation. These mutants were also defective in microtubule organization. Moreover, heterozygous mutant showed defective in-vitro yeast to hyphae morphological transition. Growth defect in heterozygous mutant suggest haploinsufficiency of this gene. qRT PCR analysis showed around 3 fold upregulation of *MPS1* in presence of serum. This expression of *MPS1* is dependent on Efg1and is independent of other hyphal regulators like Ras1 and Tpk2. Furthermore, *mps1* mutants were also sensitive to oxidative stress. Heterozygous *mps1* mutant did not undergo morphological transition and showed 5-Fold reduction in colony forming units in response to macrophage. Thus, the vital checkpoint kinase, Mps1 besides cell division also has a role in morphogenesis and oxidative stress tolerance, in this pathogenic fungus.

## Introduction

*Candida albicans*, a close phylogenetic relative of *Saccharomyces cerevisiae*, is the major fungal pathogen in humans and systemic infections are often fatal in immunocompromised patients [1]. Pathogens like *C. albicans* have evolved various counteractive cellular mechanisms to evade host defenses. Despite, these strategies some damage to key cellular components like DNA or protein of pathogen still occurs and must be repaired for survival. Cell cycle checkpoints coordinate the DNA integrity and proper chromosomal segregation during cell division which is essential for cell viability. Precise control of chromosomal segregation is carried out by a transient cytoskeletal structure termed as the mitotic spindle. The checkpoint senses defects in attachment of chromosomes to mitotic spindle thereby preventing chromosomal loss by stopping chromosome segregation. The components of checkpoint pathways are becoming drug targets, especially in cancer research. In *C. albicans*, Spindle Assembly Checkpoint pathway plays an important role in cell division [2], [3]. During nuclear division, mitotic spindle forms a bipolar structure upon the nucleation of spindle microtubules to the spindle pole body (equivalent of centrosomes in mammals) in a coordinated fashion in response to various cell division cues. In *S. cerevisiae*, seven genes have been identified that function in governing the spindle checkpoint pathway; *MAD1-3* [4], *BUB1-3* [5], and *MPS1* [6]. Further studies on Bub2 revealed that it prevents the unusual multinucleate cells with misoriented spindles from exiting mitosis. Thus, it is not a part of checkpoint, but it is one of the components that regulate the exit from mitosis [41]. Cells with a mutation in any of these genes fail to arrest the cell cycle in response to spindle defects and undergo an aberrant, lethal mitosis. A study on *MAD2* showed an indispensable role of this gene in morphogenesis and viability of *C. albicans* in murine mouse model [2]. A non-essential gene, *BUB2* controls the pre-anaphase arrest and polarization of pseudohyphal-like cells [3]. The budding yeast homolog of *CENP-A*, *CaCSE4*, has been shown to play a role in proper chromosome segregation during the growth of *C. albicans* [7].

One of the master protein kinase of check point pathway is, *MPS1* (*M*ono *P*olar *S*pindle Kinase) [6] (mutants of this gene form monopolar spindles), where the kinase activity is required for activating other members of checkpoint machinery. Homologs of *MPS1* gene have been identified in several organisms and play diverse roles in checkpoint activation, spindle pole body duplication, chromosome segregation, and mitotic arrest, in response to hypoxic conditions. In budding yeast, *MPS1* is an essential dual specificity (serine/threonine and tyrosine) protein kinase involved in the normal progression of cell cycle [6], [8]. In humans higher expression of *MPS1* have been detected in several human neoplasms, including thyroid, breast and lung cancers [9]–[12]. *MPS1* is thus considered as a promising drug target for cancer cells. Due to central role of this protein kinase in cell division, several inhibitors of *MPS1* have been reported [13]–[15]. In *C. albicans*, orf19.7293 is the *MPS1* homolog of *S. cerevisiae*. Although the inhibitor of CaMps1 is known [16] but its biological function is still unknown. In this report we have characterized a structural homologue of budding yeast *MPS1* gene and proved its indispensable role in survival, cell division, morphogenesis and oxidative stress tolerance in human pathogenic yeast, *C. albicans*.

## Materials and Methods

### Mice

Female Balb/c mouse weighing 18–20 grams were obtained as pathogen-free mice from the Animal House of Jawaharlal Nehru University (JNU) New Delhi, INDIA. The use of Mice was duly approved by the Institutional Animal Ethic committee (IAEC) of Jawaharlal Nehru University (JNU), Registration No. 19/1999 (CPCSEA; Committee for the purpose of control and supervision of experiments on animals). Approval code was VO/AH/IAEC/84/53. All housing and experimental procedures were conducted under the guidelines of the JNU Animal Care.

### Media and Strains

*C. albicans* strains used in this study are listed in Table 1. *C. albicans* was routinely grown at 30°C in YPD (1% yeast extract, 2% Bacto Peptone, 2% glucose) or Sabourad's Dextrose (SD) media (0.67% Yeast Nitrogen Base, 2% Dextrose and 2% agar (for solid media)). Synthetic Complete (SC) medium was used for conditional mutant (0.67% nitrogen base without amino acids, 2% glucose and a mixture of amino acids) and when required 80 µg ml^−1^ Uridine was supplemented (Uri+). Transformants for the HT test were grown in synthetic defined media as described by Enloe et al [17]. For conditional mutant studies, 2.5 mM L-Cystiene (Cys) and L-Methionine (Met) were externally added. For morphogenetic studies, Spider (adjusted to pH 7.3) and SLAD media were prepared as described by Lin et al [18] and Gimeno et al [19] respectively. Cells were induced with GlcNAc as per Biswas et al [20]. For serum induction 10% bovine serum (Sigma) was added to pre-warmed SD at 37°C. For G1 cell synchronization, *C.albicans* cells were cultured in Carbon source deficient Emden-Meyeroff-Mininmal Media (EMM media) at 25°C overnight [21], [22]. Chlamydospore formation was obtained by growing the cells under coverslips at 25°C for 7 days on cornmeal/Tween agar (BD) plates.

**Table 1:** Strains and plasmids used in the study.

Strain/Plasmid	Genotype/description	Source/reference
**Strains**		
SC5314 (wild-type)	*URA3*/*URA3*	
CAF3-1	Δ*ura3::imm434*/Δ*ura3::imm434*	W.A.Fonzi
BWP17	*ura3*Δ*::λimm434/ura3*Δ*::λimm434his1::hisG/his1::hisG arg4::hisG/arg4::hisG*	Aaron P. Mitchell
MFD2	As CAF3-1, but *MPS1*/Δ*mps1::hisG-URA3-hisG*	This Work
MFD2-U1	As CAF3-1, but *MPS1*/Δ*mps1::hisG*	This Work
M-UAU 1–30	As BWP17, but MPS1/Δ*Camps1::Ura3-ARG4-Ura3*	This Work
M-AU	As BWP17, but Δ*mps1::URA3*/Δ*mps1::Ura3-ARG4-Ura3*/MPS1	This Work
MCM4,7	As MFD2-U1, but Δ*mps1*/pMet*MPS1*	This Work
MCM4R	As MCM4, but pMet*MPS1*/*RP10::MPS1*	This Work
CAN52	Δ*ras1::hisG/*Δ*ras1::hph* Δ*ura3::ura3::imm434/*Δ*ura3::imm434*	[42]
HLC67	Δ*efg1::hisG/*Δ*efg1::hisG* Δ*ura3::imm434/*Δ*ura3::imm434*	[43]
AS1	Δ*tpk2::hisG/*Δ*tpk2::hisG* Δ*ura3::imm434/*Δ*ura3::imm434*	[43]
A-11-1-1-4 (Δcph1)	Δ*acpr1::hisG/*Δ*acpr1::hisG* Δ*ura3::imm434/*Δ*ura3::imm434*	Laboratory strain
**Plasmids**		
pGEM-MPS1	MPS1 ORF cloned in pGEM-T Easy vector	This work
pMP1	Carrying Δ*mps1::hisG-URA3-hisG* disruption fragment	This work
pUC19-CUB	*hisG-URA3-hisG* disruption cassette cloned in pUC19	[20]
pBME101	Carrying *Ura3′-ARG4-Ura3′* cassette	Aaron P. Mitcehll
pCaDis	Plasmid carrying MET3 promoter, URA3 marker	P.E. Sudbery
pCaEXP	Plasmid carrying MET3 promoter URA3 marker and RP10 gene	P.E. Sudbery
pCaMPF	0.5 Kb MPS1ORF with *BamH*I and *Sph*I sites cloned in pGEM-T	This work
pDMPS1	Same as pMPF with pDis vector back	This work
pCaExMP	pEXP with 2.5 kb MPS1 ORF with promoter	This work
pMUAU	Carrying Δ*mps1::Ura3′-ARG4-Ura3′* disruption fragment	This work

### Cloning, Confirmation of Essentiality and generation of conditional mutants

The entire 2073 base pairs (bp) gene was amplified from the genomic DNA of *C.albicans* (SC5314) along with 162 bp upstream and 250 bp downstream of start and stop codon respectively, using oligonucleotides, MPS1F: 5′CCTAGTGAGCACACACAT3′and MPS1R: 5′GTGTTGGCAGG GTGATGC3′ and cloned in the pGEMT-easy vector (Promega) to generate plasmid pGEM-MPS1. Following sequencing and confirmation by alignment with the Genome sequence information available at Candida genome database (CGD), the full length sequence was deposited at NCBI and an accession number was obtained (AY632356.1). First allele disruption of *MPS1* was performed using the URA blaster method [23] in CAF3-1 strain (Methods S1). One Ura^+^ heterozygous mutant (MFD2) was used for Ura-curing process, on SD+Uri agar plates supplemented with 5′-fluoroorotic acid (5-FOA) (Sigma Aldrich) at 1 mg/ml concentration. Curing was confirmed by southern blot analysis and one ura^−^ starin (MFD2-U1) was selected. A single transformation based gene function test [17] was used to confirm the essentiality of *MPS1* (Methods S1). The heterozygous MFD2-U1 strain was further used for the construction of *MET3* regulated conditional mutant, MCM4. To create *MET3*-*MPS1* cassette, a 590 bp fragment of *MPS1* coding region, comprising positions +1 to +590 in relation to the ATG start site, was PCR amplified by using oligonucleotides, MpBamHI:5′TTGAC*GGATCC*ATGCCACATGA TTTATTATC3′ and a reverse primer, MPSphI: 5′*GCATGC*ATCTTTCTAAC GACG3′, having *BamH*I and *Sph*I sites (*italicized bases*) for directional cloning. This fragment was cloned in pGEMT-easy vector which resulted in plasmid pCaMPF (Table 1). This 590 bp fragment was digested and ligated in *BamH*I and *Sph*I digested plasmid, pCaDis [24] resulting in 5,930 bp plasmid, pDMPS1 (Figure S2). This plasmid was linearized by *Bsg*I digestion and transformed into the Ura^−/−^ MFD2-U1 strain by lithium acetate method [25], to isolate *MET3* regulated strain; MCM4, on SC agar plates lacking Uridine, Methionine and Cysteine. Correct recombinants containing, *MET3* promoter was confirmed by southern blotting by digesting the genomic DNA of strains with *Xba*I and *Dra*II (Figure S2). Similarly, a rescued strain MCM4R; in which *MPS1* coding region is expressed under its own promoter, was created by reintroducing the gene at a non-essential *RPS1* locus [26] using pCaExP vector (Generously gifted by Peter E. Sudbery) [24], which contains a *URA3* marker. For construction of revertant cassette, *MPS1* ORF with 500 bp promoter region (2573 bp) was PCR amplified from the genomic DNA of SC5314 by using oligonucleotides MPSalF: 5′GTCGACTTAAATTGGTTAAAATTTTC3′ and MPSalR: 5′GTCGACATACACTAGCTGTAGTTT AAG3′ and was digested with *Sal*I enzyme. Next, pCaExP vector was digested with *Sal*I to release 360 bp *MET3* promoter fragment. The resultant 5,895 bp pCaEXP-*MET3* vector backbone was ligated with 2,573 bp *Sal*I digested *MPS1* fragment creating an 8,468 bp plasmid, pCaExMP. After linearizing by *Stu*I digestion, this construct was used for transforming MCM4 strain. Transformants were screened on SC agar plates supplemented with 2.5 mM Met/Cys, which inhibited the growth of untransformed MCM4 cells. Correct integration of pCaExMP in MCM4R was confirmed by Southern blot analysis after *Sal*I and *Dra*II digestion. Since ectopic expression of URA3 may cause phenotypic effects, so for morphological studies targeted reintegration of *URA3* was performed in the heterozygous MFD2-U1 strain using pCaEXP vector at the neutral *RPS1* locus.

### Southern blotting

For Southern blotting, genomic DNA was extracted from cells grown in YPD or SD media. five micrograms of DNA was digested with Restriction endonuclease and resolved by Agarose gel electrophoresis on 1% agarose gels, before transferring [27] them to positively charged Nylon membranes (NEN Research Products Ltd.,) by capillary transfer. For screening of MFD2, MFD2-U1,M-UAU and M-AU transformants hybridizations were performed with ^32^P radiolabelled DNA probes, prepared from *Not*I-digestion of pGEM-MPS1 that resulted in a ∼2.5 Kb *MPS1* fragment. Whereas a 0.597 Kbp fragment of pDMPS1, which was digested by *BamH*I & *Sph*I was used for screening of *MET3* transformants.

#### qRT-PCR

Cells were grown to an OD 0.8 in SD media [28] and induced for two hrs in synthetic media with 2% carbon source. Total RNA was isolated using Tripure reagent (Roche). cDNA was synthesized from total RNA (5 µg) using an oligo dT primer (Invitrogen) and Superscript I reverse transcriptase (Invitrogen). The original mRNA template was removed from the RNA: DNA heteroduplex with RNase H. The cDNA samples were then used as templates for qRT-PCR in a 7900 Fast Real Time PCR system (Applied Biosystems) using SDS 2.4 program. The 10 µl reaction mix included 2X KAPA SYBR FAST (KAPA BIOSYSTEMS), 0.5 µl of cDNA and 0.2 µM primers. The amplification program included Stage I- 50°C for 2 min, Stage 2- 95°C for 10 min, Stage 3- 40 cycles of 95°C for 15 sec and 60°C for 60 sec, Stage 4- 95°C for 15 sec, 60°C for 15 sec and 95 for 15 sec. Raw data was analyzed using RQ manager 1.2.1 software to determine relative gene expression. *ACT1* mRNA level was used as endogenous control. The results represent the average of three biological replicate experiments each performed in triplicates. Error bars, where ever exist represent the standard error between 3 separate biological replicates. The following primers designed with Primer 3 software were used for qPCR analysis. *ACT1*-F5′ GACAATTTCTCTTTCAGCACTAGTAGTGA -3′ and *ACT1*-R5′ GCTGGTAGAGA CTTGACCAACCA -3′; *MPS1-F*5′ CCAGGCAGCAAAACATT *3*′*MPS1-R*5′ TTTGCCGAACCTTCTTT *3*′.

### Indirect Immunofluorescence

Cells were grown up-to an OD of 0.6–0.8 in SC medium at 37°C and conditional mutants were induced with 2.5 mM Met/Cys for 4 hrs. Cells were fixed with 4% formaldehyde for 30 min at room temperature and washed four times with PBS (150 mM NaCl: 20 mM Na/K phosphate, pH 7.3). Cells were resuspended in PBS containing 1 M sorbitol (PBSS) and 100 µl of zymolyase and incubated at 37°C for 1 hr. Cells were washed twice with PBSS and resuspended in 100 µl PBS +1% BSA +0.1 M lysine and cell were further added to the slides and allowed to stand for 30 min. Cells were then permeabilized with chilled methanol (9 min) and acetone (6 min), and air-dried. After rehydration in PBS, slides were incubated with the anti-tubulin antibodies (Millipore, CBL270) diluted 1∶200-1∶400. Antibodies for immunofluorescence were diluted in PBS, 1% BSA, and antibody incubations were carried out for 1 h at 37°C. The slides were washed for 20 min with four changes of PBS, and then the second antibody FITC conjugated goat anti Rat IgG was used at 1∶200 dilution. Finally, the slides were washed for 20 min with four changes of PBS mounted and viewed under confocal microscope.

### Nocodazole Sensitivity Assay

Strains were grown up-to an OD of 0.6–0.8 in liquid SD media and induced with Nocodazole (50 µM/ml) (Sigma aldrich). Aliquots of treated cultures were collected at 2, and 4 hr intervals. Conditional mutants were induced with Nocodazole either in presence or absence of 2.5 mM Met/Cys. For viability check, cultures were serially diluted and equal numbers were spreaded on SD Agar plates and incubated at 30°C for 2–3 days. For S-phase arrest, cultures were treated with 50 mM Hydroxy Urea (USB biochemicals) at 30°C for two hours before the addition of nocodazole. Cells were collected and plated as described earlier [2].

### Cell Synchronization and Flowcytometry

*C. albicans* cells were prepared for FACS analysis by Nitrogen starvation based synchronization as described [21]. Cells were grown in Edinburgh Minimal Medium (EMM) till an OD of 0.5. Cells were then inoculated in EMM medium without Ammonium Chloride and grown to saturation at 23°C with vigorous aeration. At regular intervals, cells were harvested and inoculated in SD or SC medium supplemented with nocodazole or hydroxy urea. Fixation was done by 70% chilled ethanol and incubated for one hour at 25°C. After fixation, cells were harvested and washed twice with 5X Tris-EDTA buffer (pH 8.0). Further, cells were treated with 1 mg/ml RNaseA (Invitrogen) at 37°C for three hours. Washed with PBS and stained with 50 µg/ml Propidium Iodide at 4°C for 8–10 hrs. Stained cells were diluted five folds and briefly sonicated to disperse aggregates formed during fixation process. Approximately 1–2×10^4^ cells was analyzed by BD FACS Calibur machine by gated settings on a linear scale. Forward Scatter analysis (FSC) was performed in a similar way. Histograms were processed and analyzed by Morphid and WinMDI softwares, respectively.

### Morphogenetic studies in mouse peritoneal cavity and macrophages

Freund's complete adjuvant (0.4 ml) was injected into the peritoneal cavity of female Balb/c mouse, weighing 18–20 grams. 10 days after the injection, a significant abdomen enlargement was observed due to large amount of exudates in the peritoneal cavity. Further, 2×10^8^ late log phase cells of each strain in 200 µl of PBS was injected into enlarged peritoneal cavity (three mice for each strain were used). 24 hours after the injection, 100 µl of the peritoneal exudate was retrieved using a 23G hypodermic needle attached to a 1 ml syringe. Peritoneal exudates were smeared on microscopic glass slides and stained as described in [2]. Photographs were taken with a Leica digital camera attached to the microscope.10 µl of peritoneal exudates was added to 190 µl sterile MQ for lysing the macrophages and the solution was plated on YPD agar and incubated at 37°C for 3 days to determine the *C.albicans* colony forming units (CFU).

### H_2_O_2_ sensitivity

Cells were grown upto an OD of 0.8 at 30°C in SD media and treated with different concentrations of H_2_O_2_ for 1 hour. Cells were further spotted on H_2_O_2_ free SD agar plates and incubated at 30°C for two days to determine the effect of H_2_O_2_.

## Results

### In silico analysis of putative *MPS1* in *C. albicans*

**Figure 1:**
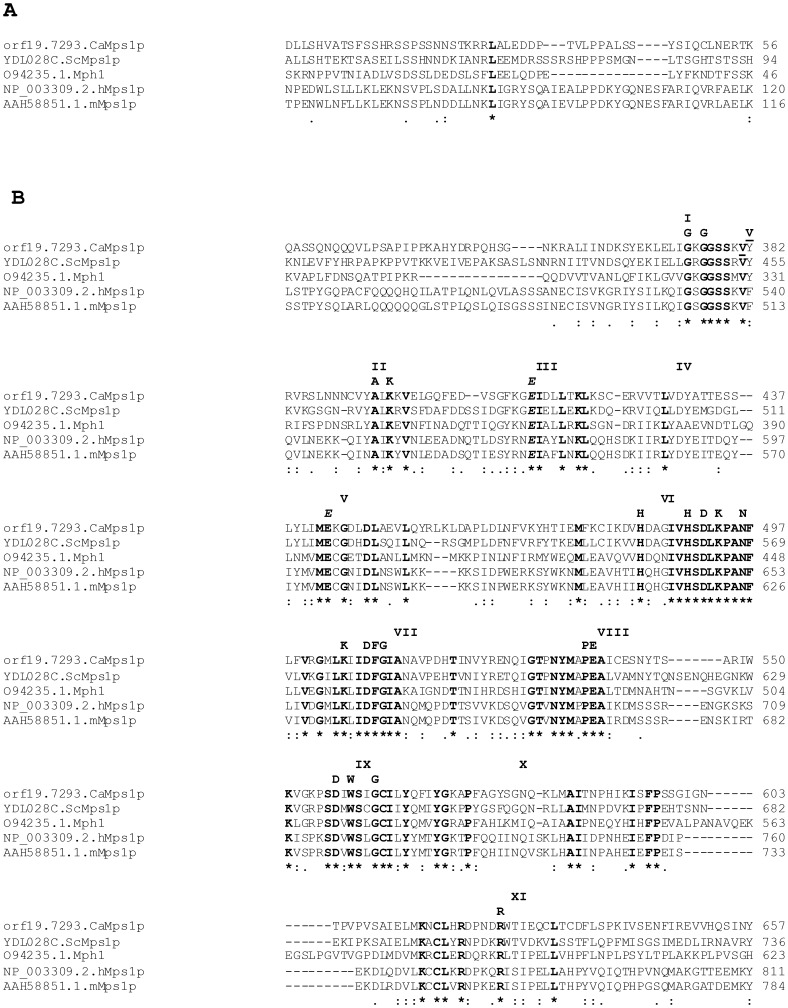
Multiple sequence alignment showing conserved regions of Mps1p homologs. (**A**) Alignment of N-terminal region of Mps1, showing conserved Leucine residue. (**B**) Sequence alignment to show C-terminal homology of CaMps1 (Orf19.7293/AY632356), Mps1 of *S. cerevisiae* (YDL028C/P54199), Mph1 of *S. pombe* (O94235), hMps1 of Human (Q9BW51) and mMps1 of Mouse (P35761): Where ORF numbers were not obtained, accession numbers are provided. Eleven subdomains are shown in Roman numerals (I–XI). Subdomains of the kinase domain are shown as described by [30]. The conserved amino acids in serine/threonine kinases (SER/THR) are shown as described by [31], conserved amino acids are shown in bold letters. The italicized ***E*** in the sub domain V is not part of the SER/THR consensus, but a feature of dual specificity kinases [31]. Valine (V) at position 382 is the site of insertional inactivation. Well conserved aa are indicated with a double dot (:) and the perfectly conserved aa are indicated with an asterisk (*)

Genes of *MPS1* family are highly conserved among eukaryotes. Orthologs of this gene are present in majority of the organisms, with the exception of *Caenorhabditis elegans*. We cloned the 2073 bp putative *C.albicans MPS1* homolog of *S. cerevisiae*, using the gene sequence provided by Candida Genome Database (CGD) [29]. CaMps1 encodes a protein of 690 amino acids with an estimated *M*r of 77.69 kDa. A Clustal W multiple sequence alignment (http://www.ebi.ac.uk/) was used to determine the homology of putative CaMps1, with the characterized Mps1 from *S. cerevisiae*, *S. pombe*, *Mus musculas* and Human (Figure 1). CaMps1 shared a significant homology of 67% at protein and 54% at DNA sequence level with the *S. cerevisiae* Mps1. Multiple sequence alignment showed that the C- terminal region of CaMps1 has all eleven conserved sub domains (from amino acids 367 to 637) characteristic of Serine/threonine kinase domain of Mps1 [30] (Figure S1A). CaMps1also has one tyrosine kinase specific signature, glutamine (E) residue in the sub domain V (at position 443) (Figure 1) [31]. The kinase function of Mps1 family is known to be associated with processes such as chromosome segregation, genome stability and other vital cellular processes [32]. Phylogram revealed that, CaMps1 clustered with *D. hanseii*'s predicted Mps1 sequence and has closeness to ScMps1 and *Candida glabrata* Mps1 (Figure S1B). ScMps2 and ScMps3 formed out-group in the phylogram indicating evolutionary divergence as compared to the members of Mps1 family of kinases (Figure S1B). Apart from CaMps1, Rad53 (cell cycle checkpoint) and Orf19.3459 (an ortholog of *S. cerevisiae* Mck1p) also have dual specificity kinases signatures in *C. albicans*.

### Disruption of *CaMPS1* gene

**Figure 2:**
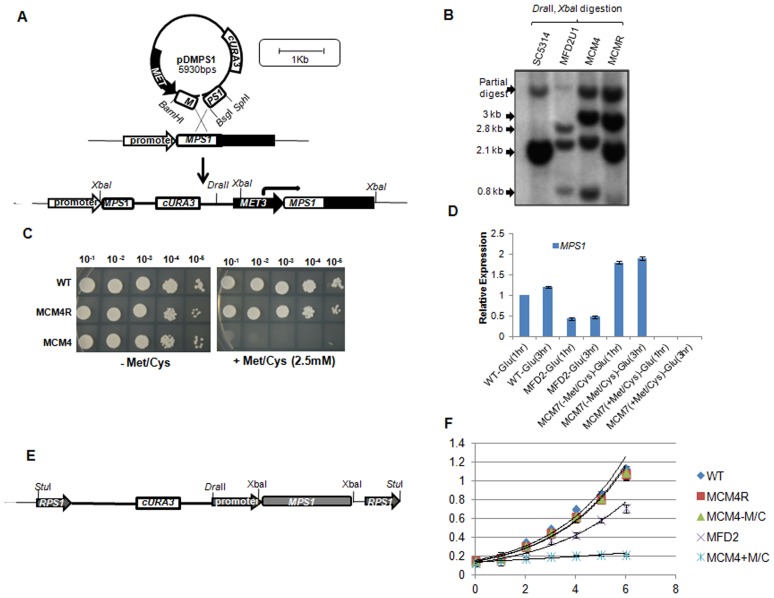
Construction of *MET3* promoter regulated conditional *mps1* mutant. (**A**) Illustration representing conditional mutant preparation. Plasmid pDMPS1was digested with *Bsg*I and linearized cassette was used to transform the *URA3*-cured MFD2 (*MPS1/mps1*Δ*:hisG*) mutants. Genetic structure of MCM4 locus after homologous recombination is represented. (**B**) Southern blot analysis to confirm promoter replacement. Genomic DNA of strains was digested by *Dra*II and *Xba*I which resulted in a 2.8 Kb and 3 Kb bands in MCM4 strain, where as 2.1 Kb band is the part of Wild type and MCM4R strain. MFD2-U1 is the URA-cured first allele mutant. Complete ORF of the gene was used as probe. (**C**) Spot dilution assay to Check the growth response of conditional mutants in absence (−) or presence (+) Met/Cys containing Complete Synthetic medium (SC Agar). Mid log phase grown cultures were serially diluted and spotted. Plates were incubated at 30°C for 2–4 days. (**D**) qRT-PCR analysis to compare the expression of *MPS1* in wild-type (WT), heterozygous (MFD2) and conditional mutant.

Mps1 family of protein kinase play a role in spindle checkpoint activation in majority of the organisms studied. For determining the function of *CaMPS1* gene in *C. albicans*, we created the knockout. Though *MPS1* gene is essential in most of the organisms including *S.cerevisiae*, but *MPS1* homolog (*MPH1*) of *Schizosaccharomyces pombe* is non-essential [33]. To confirm the essentiality of *MPS1* gene in *C. albicans*, we adopted a specially designed single transformation based gene function test called Homozygote Trisome test. Southern blot analysis revealed that all independent Arg+ Ura+ segregants were *mps1*::*UAU1*/*mps1*::*URA3*/*MPS1* triplication derivatives (Figure S2D). Thus, presence of only allelic triplication confirmed that *MPS1* is an essential gene. Furthermore, we created the conditional mutants by replacing promoter region of *MPS1*, in the heterozygous MFD2 strain (*mps1/MPS1*), with the methionine (Met)/Cysteine (Cys) suppressible *MET3* promoter (Figure 2A). Two positive strains, MCM4 and MCM7 (Table 1) were selected for functional analysis and their promoter replacement was confirmed by southern blot analysis (Figure 2B). A serial dilution spot assay showed that MCM4 strain (Δ*mps1*/pMet*MPS1*) did not grow in presence of 2.5 mM Met/Cys suggesting conditional inhibition of growth in this strain (Figure 2C). No change was observed in the growth of MCM4 strain in absence of Met/Cys (Figure 2C). The expression levels of *MPS1* were also determined under the *MET3* promoter (Figure 2D). qRT PCR analysis showed that in comparison to native promoter, *MPS1* expression was a little higher under the influence of *MET3* promoter in absence of Met/Cys. In presence of Met/Cys no expression of *MPS1* was observed. An additional copy of *MPS1* under its own promoter was introduced into MCM4 strain within non essential *RPS1* gene (Figure 2E). Correct integration was confirmed by Southern blot analysis (Figure 2B). Growth rate studies in liquid SD media showed that conditional mutant in absence of Met/Cys behaved like wild type strain, but in presence of Met/Cys did not show any growth. The heterozygous mutant (MFD2) showed delayed growth as compared to that of wild type strain (Figure 2F). Growth of revartant strain (MCM4R) having additional copy of *MPS1* at neutral *RPS1* locus was also analyzed. No significant change was observed in the growth of MCM4R strain as compared to wild type.

### Role of *MPS1* in normal ploidy maintenance

**Figure 3:**
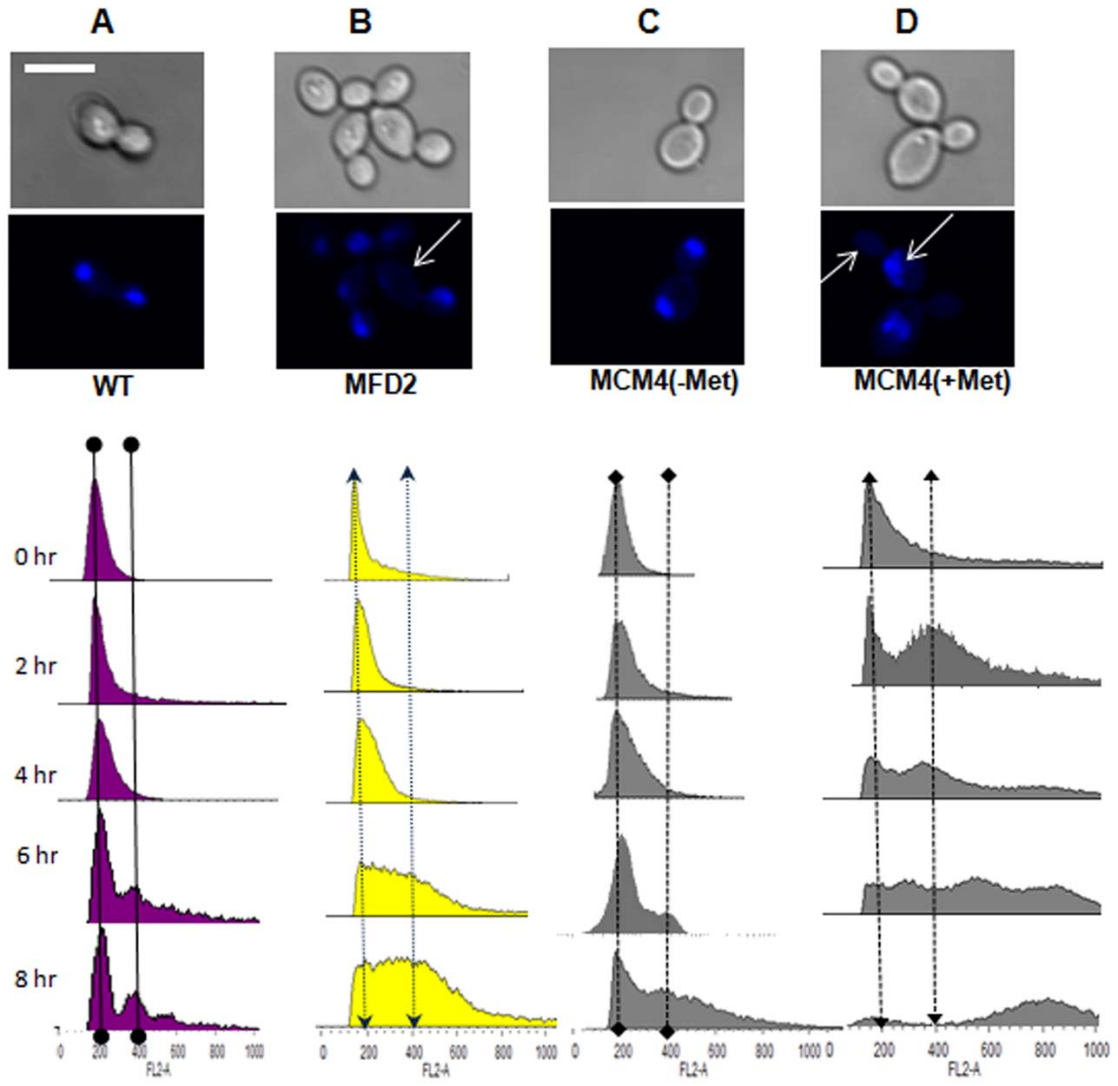
Fluorescence and flowcytometry analysis of wild type (WT) and *mps1* mutant strains. For studying the defects in nuclear division and chromatin segregation in *mps1* mutants, cells were stained with DAPI after 4 hours of growth in liquid SC medium at 37°C. WT (**A**), heterozygous mutant (MFD2) (**B**), conditional mutant –Met/Cys (**C**) and conditional mutant +Met/Cys (**D**). Arrows indicate defective chromosome segregation. For flowcytometry analysis, chromatin was stained with Propidium Iodide, at indicated time points. The X-axis indicates the amount of DNA measured and Y-axis represents number of cells. Approximately, 10^4^ cells of each strain were analyzed on a FACS-Calibur machine on FL2-A channel. Two imaginary lines were drawn to represent changes in position of peaks of 2 N and 4 N content of DNA, respectively. Scale bar shown is10 µm and is applicable to all images.

Mps1 is known to be a master protein kinase, which phosphorylates the downstream molecules of spindle checkpoint pathway, like Mad1/Mad2 and Cenp-E [32]. In budding yeast, mutants of these genes displayed defects in chromosome segregation, distribution of nuclei and ploidy levels [2], [34]. Fluorescence microscopy of 4′, 6′-diamidino-2′-phenylindole (DAPI) stained *mps1* mutant strains, revealed that around 58% of the heterozygous cells (MFD2) had large buds without nuclei (Figure 3B) (Table 2). MCM4 strain in absence of Met/Cys behaved as wild type but, in presence of Met/Cys the cells were larger, on average, for both cell and nuclear size as compared to wild type and around 70% cells had bud without nuclei (Figure 3D) (Table 2).This suggested that CaMps1 could be required for proper chromosomal distribution during mitosis. Growth defects in single allele knockout indicates haploinsufficiency of this gene [35]. *MAD2* gene of *C. albicans* and mammals also show haploinsufficiency, where heterozygous mutants displayed premature anaphase and chromosome instability [2], [36].

**Table 2:** Comparison between wild type and Δmps1 strains with respect to observed phenotypes via DAPI staining.

Time	Strain	Percentage of non dividing cells	Percentage of dividing cells with buds without nuclei	Percentage of dividing cells with normally dividing nucleus	Percentage of mother cell and bud with nuclei	No. of cells counted
2Hr	Wild Type	94	1	1	4	226
	MFD2	40	38	7	15	250
	MCM+M/C	25	55	8	12	278
	MCM-M/C	92	2	1	5	245
4Hr	Wild Type	83	1	2	14	291
	MFD2	27	58	6	9	282
	MCM+M/C	21	67	8	4	274
	MCM-M/C	76	2	3	19	265

Cells were stained with DAPI and counted on hemocytometer. Cells were grown in SD media at 30°C for indicated time points. Average percentages of three biological replicates are shown in the table.

**Figure 4:**
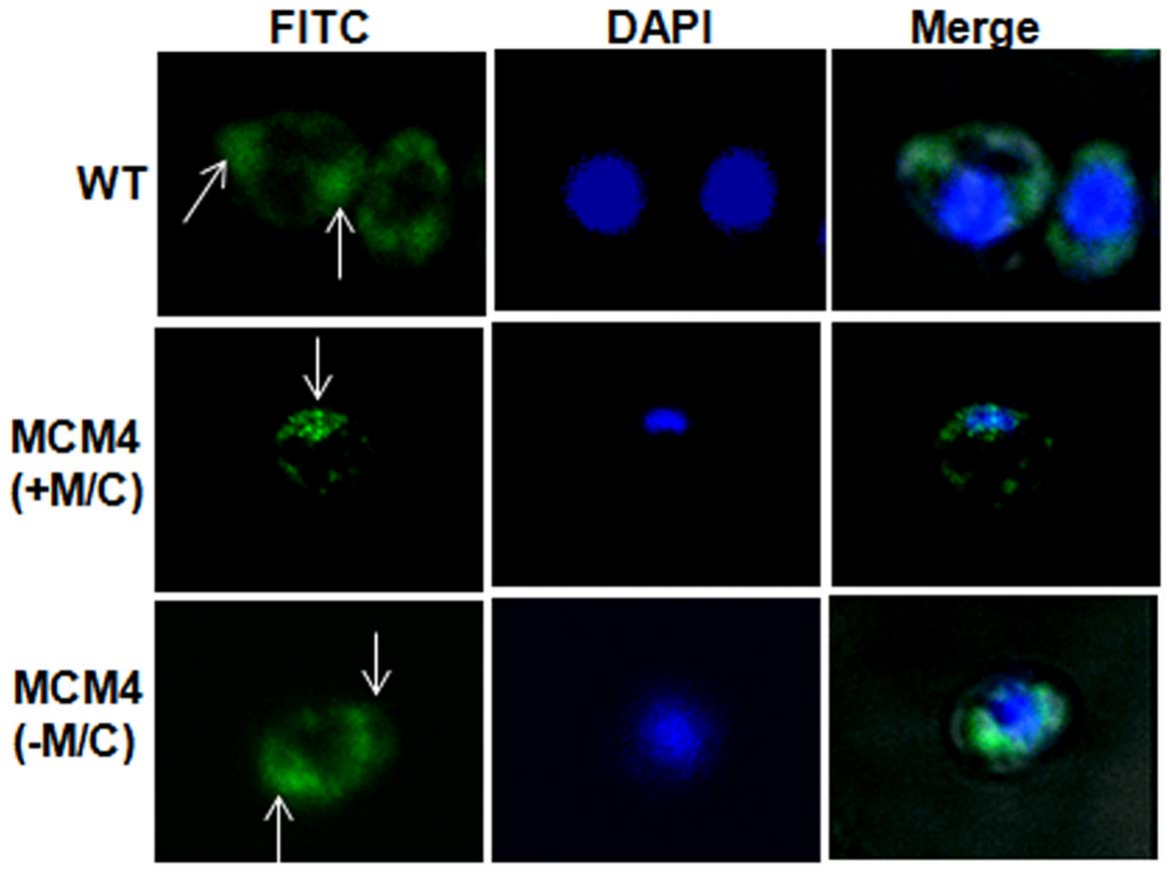
Immunostaining with anti α- tubulin antibodies. Conditional mutant in presence of 2.5 mM Met/Cys showed formation of mono polar spindles. Wild type (WT) and conditional mutant in absence of Met/Cys showed proper bipolar spindles. White arrows indicate position of spindles (Green fluorescence) in the strains.

To investigate the role of Mps1in chromosome segregation, we quantitatively analyzed the DNA content of mutants by Propidium Iodide staining based flowcytometry assay. On inducing the MCM4 strain in a medium containing Met and Cys, we observed a progressive shift in DNA content, from 2 N to 4 N and 8 N (Figure 3D) which indicated accumulation of more DNA per cell than that in similarly treated control cells (Figure 3, A and E). However, MCM4 strain in absence of Met/Cys, continued to grow with 2 N content of DNA per cell (Figure 3C), though after prolonged growth (8 hrs) cells with 4 N were observed. Thus, fluorescence microscopy and flowcytometery analysis of *MET3* conditional mutants, suggest a role of Mps1 in normal maintenance of ploidy and the genomic stability of *C. albicans*. Analysis of microtubule organization was also performed through immunostaining for α-tubulin. Wild type and conditional mutant (-Met/Cys)) cells displayed the normal bipolar spindle organization, where the spindles appear to be originating from the opposite end of the cells (Figure 4). In contrast, microtubules in the conditional mutant (+Met/Cys) were present at one end of the cells, indicative of cells having monopolar spindles (Figure 4). Thus, defective microtubule organization is the reason for inaccurate chromosomal segregation in *mps1* mutants as determined by DAPI staining.

### *MPS1* is required for the activation of spindle checkpoint pathway

**Figure 5:**
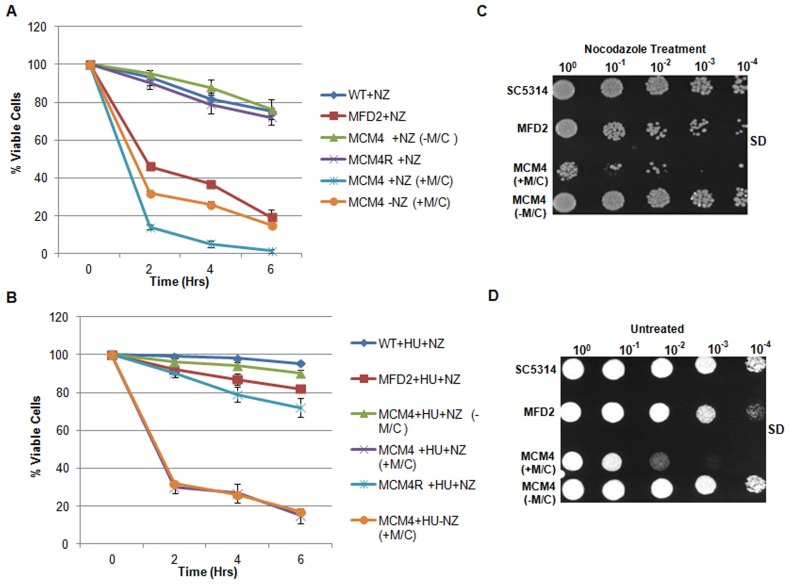
Sensitivity of *MPS1* mutants to nocodazole. (**A**) Exponential phase yeast cells were treated with 50 µM Nocodazole at 30°C and aliquots were collected after 2, 4 and 6 hours to determine CFU on Nocodazole free SD agar plates. (**B**) Cultures were first treated with hydroxyurea for 2 hrs before the addition of Nocodazole; aliquots were collected for counting CFU. (**C**) Cells were treated with Nocodazole (50 µM/ml) for 6 hrs and plated on Nocodazole free plates following washing and serial dilution for 2 days at 30°C. **D**) Untreated control cells were spotted on SD agar plates for 2 days at 30°C.

Mps1 family of protein kinases in budding yeast and other organisms have roles in the activation of Spindle Assembly Checkpoint (SAC) pathway and spindle pole body duplication [32]. To determine any loss of SAC function in *mps1* mutants, both heterozygous and conditional *mps1* mutant strains were exposed to the microtubule toxin, Nocodazole. Cells were treated with 50 µM Nocodazole for 2, 4 and 6 hours and harvested to determine colony forming units (CFUs) on SC-Met/Cys agar plates (Material and methods). The results showed that with increase in time, conditional mutants in presence of Met/Cys, readily lost viability in presence of Nocodazole as compared to untreated cells (Figure 5A). Moreover, before treatment with Nocodazole hydroxy-urea treatment was also given to arrest the cells in S-phase of cell cycle by inhibiting the ribonucleotide reductase. Under this condition, cell mortality was minimized, where ∼95% of wild type and ∼85% of heterozygous mutant cells retained the viability (Figure 5B). This showed that microtubule disruption by Nocodazole alone is not responsible for cell death, but indeed it was due to passage through the cell cycle in absence of SAC function, caused the cell death in *mps1* mutants in response to Nocodazole.

Additionally, a serial dilution spot assay also indicated loss of growth in presence of Nocodazole (Figure 5C) as compared to untreated control plate (Figure 5D). Both heterozygous and conditional mutants (+Met/Cys) showed sensitivity to Nocodazole treatment (6 hr). It was also observed that even in absence of Nocodazole the Met/Cys treated conditional mutant showed loss of viability (Figure 5D). This could be due to essential role of Mps1 in cell division. Thus, a rapid fall in the viability of Nocodazole treated conditional mutant in presence of Met/Cys could be attributed to the dual action of microtubule disruption and suppression of *MPS1* expression. Conditional mutants in absence of Met/Cys behaved as wild-type. These results demonstrate a role of Mps1 in the activation of SAC in *C.albicans*.

**Figure 6:**
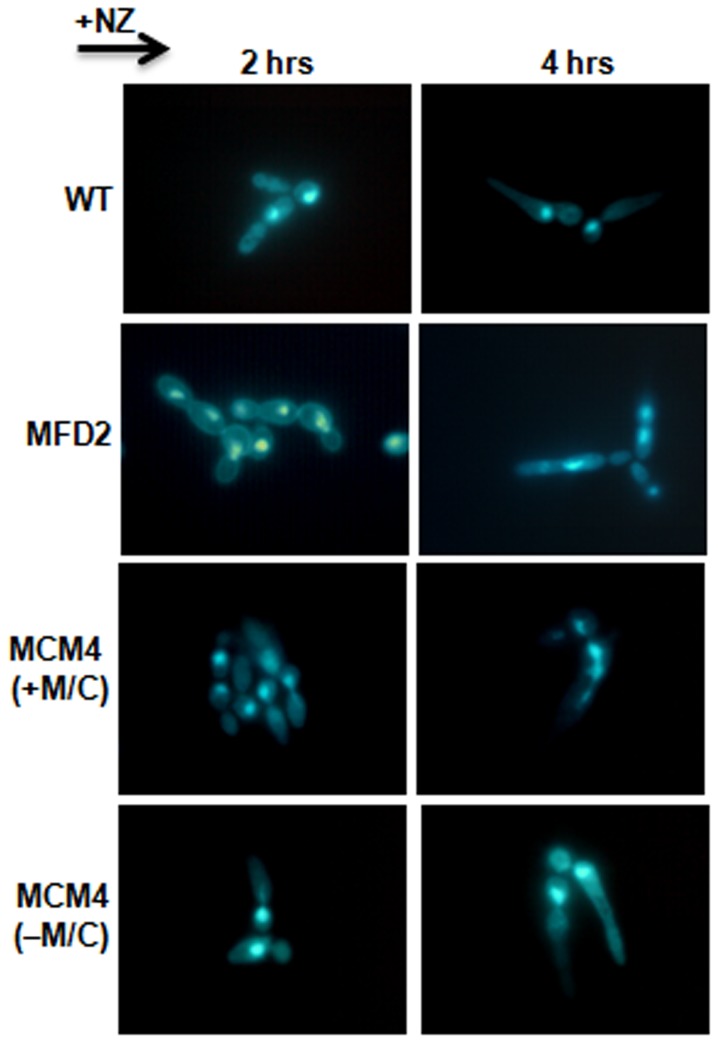
Polarized growth study and nuclear dynamics in nocodazole treated cells. Exponential phase cells of indicated strains were treated with Nocodazole (50 µM/ml) in liquid SD media at 30°C and harvested at indicated time points and stained with DAPI.

The morphogenetic response of *C. albicans* to checkpoint inducing drugs like Nocodazole is quite different from that of budding yeast, *S. cerevisiae* where yeast cells either remain largely budded or have multiple buds [37], [38]. In *C.albicans*, conditions that arrest cell cycle progression often result in a polarized growth phenotype [38], [39]. To determine the morphological response, DAPI staining and fluorescence microscopy were performed after treating cells with 50 µM nocodazole. It was observed that after 2 hrs of drug treatment nearly all wild type cells formed elongated buds, without nuclei indicating cell cycle arrest (Figure 6). After 4 hr, wild type cells showed pseudo-hyphal like extensions, with single nucleus present either in mother cell or elongated bud. The heterozygous and conditional mutant (MCM4) in presence of Met/Cys showed buds of different sizes suggesting absence of cell cycle arrest (Figure 6). Conditional mutant in absence of Met/Cys behaved like wild type (Figure 6). After 4 hours heterozygous and conditional mutant cells showed irregular shape and were often multinucleate (Figure 6). Thus, Mps1 plays an important role as the cell cycle checkpoint, but it is not involved in regulating the Nocodazole induced polarized growth.

### Role of Mps1 in morphogenesis

**Figure 7:**
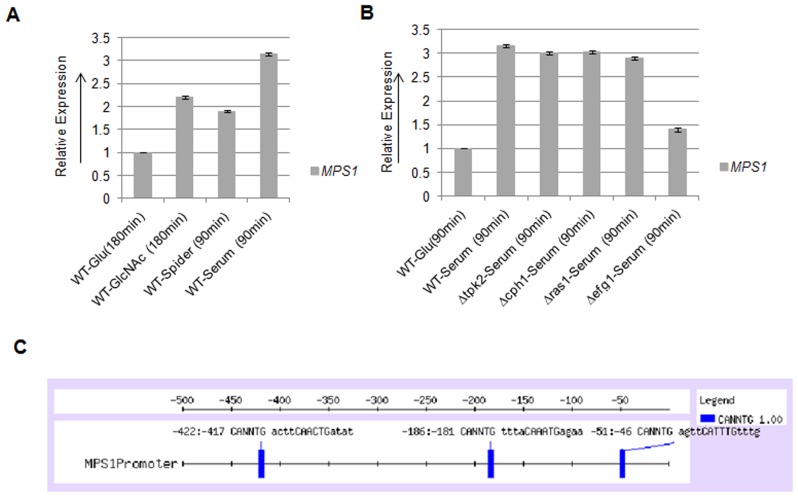
Expression analysis of *MPS1*. **A**) qRT-PCR analysis to determine the expression of *MPS1* in indicated hyphae inducing media. **B**) qRT-PCR to determine the regulation of *MPS1* expression in presence of serum. **C**) Promoter analysis of *MPS1* gene: 500 bp of upstream sequence of *MPS1* was analyzed by using Regulatory Sequence Analysis tools (RSAT) server at, http://rsat.ulb.ac.be/rsat. Efg1p binding element 5′CANNTG3′ was identified at three locations.

*C. albicans* is a polymorphic organism and exhibits variety of cellular forms. The ability of this organism to switch between distinct modes of proliferation contributes towards its virulence. We investigated the role of Mps1 in the yeast to hyphae morphological transition of *C. albicans*. qRT-PCR analysis showed higher expression of *MPS1* gene in major filamentation inducing conditions like, GlcNAc, spider and serum as compared to that of Glucose containing media (Figure 7A). Maximum expression of *MPS1* was observed in 10% Serum (3 folds) followed by GlcNAc (2 folds) as compared to glucose grown cells.

To identify the signaling network required for the expression of *MPS1* gene, qRT PCR analysis was performed on mutants of major signal transduction pathways that control morphogenesis. This analysis showed that the expression of *MPS1* was unchanged in Δ*tpk2,* Δ*cph1* and Δ*ras1* mutants, but in Δ*efg1* mutant the expression was suppressed to basal level (Figure 7B). Thus, the expression of *MPS1* is dependent on cAMP mediated signaling pathway through Efg1, but independent of both Tpk2 and Ras1. Computational analysis of *MPS1* promoter, showed three Efg1 binding elements (CANNTG) within 500 base pairs upstream of start codon, at positions −46, −181 and −417 (Figure 7C).

**Figure 8:**
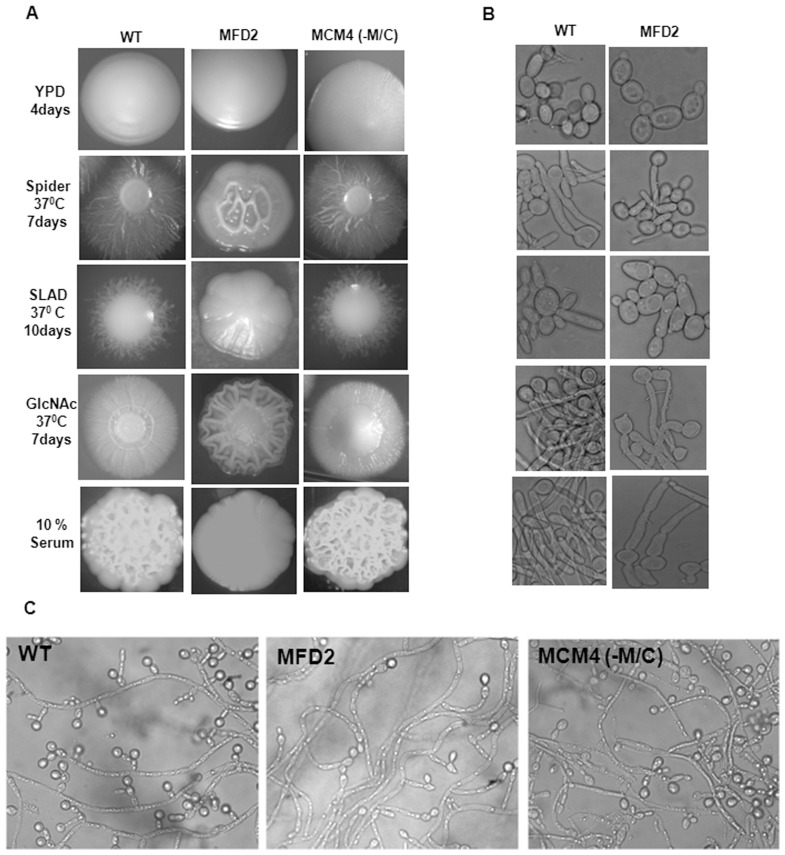
Morphology of *mps1* mutant strains under different hyphae inducing conditions. (**A**) Morphogenetic studies on solid inducing media: Cultures under study were grown to mid log phase, serially diluted and 25 to 50 cells per plate were spreaded on indicated hyphae inducing media. (**B**) Morphologies in respective liquid inducing media; yeast cells of overnight cultures were inoculated into indicated inducing media at 2×10^5^ cells/ml for 3 hours. (**C**) Chlamydospore formation as observed after 7 days at 25°C on cornmeal/Tween agar plates under coverslips.

Higher expression of *MPS1* in major hyphae inducing conditions suggests its role in morphogenesis. So, we analyzed the morphological transition of *mps1* mutants on various solid and liquid filamentation inducing media like Spider, SLAD, GlcNAc and 10% Bovine Serum. The heterozygous strain (MFD2) showed defective filamentation on all solid agar media that were tested (Figure 8A). The conditional mutant (MCM4), in absence of Met/Cys behaved as the wild type strain (Figure 8A). Like on solid media, in liquid inducing media also, heterozygous mutant cells have a reduced response for yeast to hyphal transition even after prolonged incubation (Figure 8B). Morphologically, MFD2 cells were elongated, swollen and formed pseudo hyphal like growth without any true hyphae. The morphology of conditional MCM4 strain in absence of Met/Cys and rescued strain (MCM4R) in liquid culture was comparable to wild type in all media tested (data not shown).

One of the distinctive morphological features of *C. albicans* is formation of Chlamydospores under oxygen limited environment. So, role of Mps1in chlamydospore formation was also analyzed. Strains were grown for 10 days in dark at 25°C under microaerophilic conditions. Large numbers of chlamydospores were observed in wild type strain, situated at the end and branching of the filaments, but the heterozygous mutant showed sparse and elongated branching of filaments with significantly reduced chlamydospores at end of filaments (Figure 8C). No change in the chlamydospore formation was observed in heterozygous strain even after prolonged incubation of 21 days. Conditional mutant in presence of Met/Cys showed a response similar to wild type (Figure 8C).

### Sensitivity of *mps1* mutants to oxidative stress

**Figure 9:**
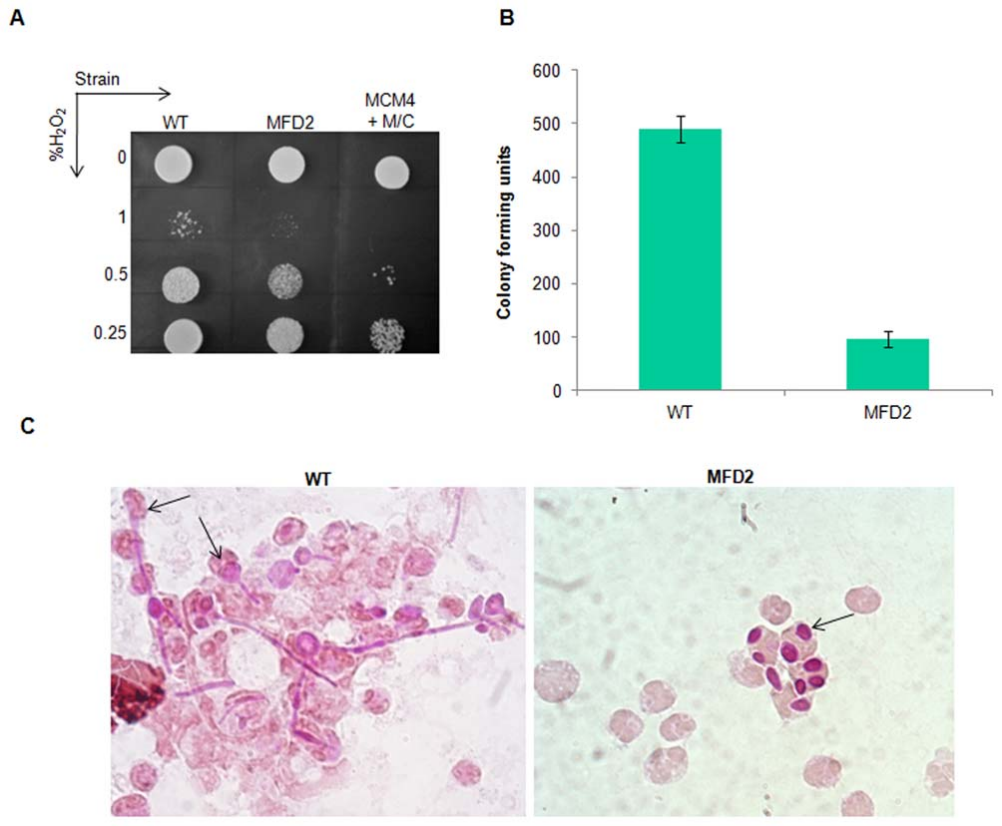
Oxidative stress tolerance of *mps1* mutants. **A**) Mutants were cultured in Liquid SD medium till mid log phase and resuspended in PBS (pH 7.4) containing indicated concentrations of H_2_O_2_ and treated for 1 hour at 37°C. Cells were spotted upon serial dilution on H_2_O_2_ free SC-Agar plates and incubated at 30°C for 2 days. (**B**) 10 µl of peritoneal exudates of mice injected with indicated strain was added to 190 µl sterile MQ and plated on YPD agar plates to determine CFU. Error bars represents standard error between three biological replicates. (**C**) Morphology of wild type (WT) and heterozygous *mps1* mutant strains injected into the peritoneal cavity of mouse. Peritoneal exudates were retrieved after 24 hrs of injection. *C. albicans* cells of indicated strains engulfed by macrophages were stained with PAS stain. Arrows indicate *C. albicans* cells engulfed by macrophages.

One of the important ways by which macrophages of host immune system defend endocytosed pathogens, is by the release of reactive oxygen species. So, pathogens have evolved mechanisms required for protecting themselves from such free radical effects. It was previously demonstrated that, checkpoint proteins are required for encountering such free radical attacks caused by macrophages [2]. Since CaMps1is required for the activation of spindle checkpoint pathway, we analyzed its involvement in oxidative stress tolerance. Thus, *mps1* mutants were treated with H_2_O_2_ (60 min) and spotted on H_2_O_2_ free SC agar plates, to facilitate the growth of cells which escaped the free radical attack. After 2 days of incubation at 30°C, *mps1* mutant strains showed sensitivity towards H_2_O_2_ as compared to that of wild type strain (Figure 9A). The conditional mutant MCM4 incubated with H_2_O_2_ in presence of Met/Cys showed much higher sensitivity as compared to heterozygous strain. This result demonstrates the role of Mps1 in oxidative stress tolerance in *C. albicans*.

Since *MPS1* gene is required for oxidative stress response, it is particularly important to check their response in macrophages, where oxidative free radical attack is a first hand of defence. To examine this heterozygous mutant and control wild type strains were injected in to the peritoneal cavity of mice. Cells were subsequently retrieved from the peritoneal exudates after 24 hours of injection. Survival of the heterozygous mutant (MFD2) within the macrophages was determined by plating the exudate on YPD agar and counting the colony forming units (CFU) of the strains. A 5-fold decrease in CFU was observed in the MFD2 strain in comparison to the wild type (WT) strain (Figure 9B). This could be attributed to the sensitivity of MFD2 strain to oxidative stress on exposure to macrophages. Since, hyphae formation in *C. albicans* is required for rupturing the macrophages to facilitate escape from the hostile environment of the phagosome. A microscopic examination of peritoneal exudates was performed for examining the morphological transition on exposure to macrophages under *in vivo* conditions. After 24 hrs of exposure to macrophages the wild type strain, engulfed by macrophages showed significant filamentation (Figure 9C). On the contrary, MFD2 strain, engulfed with macrophages showed only yeast form (Figure 9C). Thus, heterozygous *mps1* mutant failed to undergo morphological transition even in response to macrophages.

## Discussion

In microbial pathogens like *C. albicans* the spindle checkpoint machinery play an important role in survival within host. Because these organisms grow under the constant threat of host defense mechanisms, so damage to cellular components like DNA is inevitable. The checkpoint machinery ensures proper chromosomal segregation. Deregulation of this checkpoint machinery leads to aneuploidy and chromosomal instability. In this report, we have characterized the *S. cerevisiae* Mps1 homolog in *C. albicans*. Essentiality of *MPS1* in *C. albicans* was confirmed by Homozygote Trisome test. To study the function of the gene, we created the conditional mutants by replacing the promoter of *MPS1* with Methionine/Cysteine regulatable *MET3* promoter. Analysis of *mps1* mutants showed that under normal conditions this gene is required for proper segregation of chromosomes. When mutant cells were stained with DAPI, they often displayed a single, largely stained nuclear region with buds without nucleus, suggesting that mutant cells have failed to complete nuclear division. Flowcytometry analysis also showed the increase in ploidy levels of *mps1* mutants with time. This particular feature is a reminiscent behavior of the *mps1-1* mutants of budding yeast [6].

In eukaryotes the spindle assembly checkpoint is highly conserved. It monitors the attachment between kinetochores and microtubules during prometaphase. Under conditions where kinetocores lack proper tension or lack of microtubule occupancy at kinetochore, it halts the metaphase to anaphase transition. Several genes of *S. cerevisiae* and *C. albicans* share evolutionarily conserved functions. In *S. cerevisiae* Mps1 play an important role in SAC. So, we analyzed whether SAC function of Mps1 is conserved in *C. albicans*. We adopted the Nocodazole sensitivity assay to find the role of *MPS1* gene in spindle checkpoint activation. In the presence of Nocodazole, mutants readily lost their viability, suggesting that, this gene is required for normal activation of spindle assembly checkpoint (SAC) pathway. Moreover, Nocodazole treated mutants cells also showed absence of cell cycle arrest. This suggests that SAC function is well conserved in *C. albicans* Mps1.

**Figure 10:**
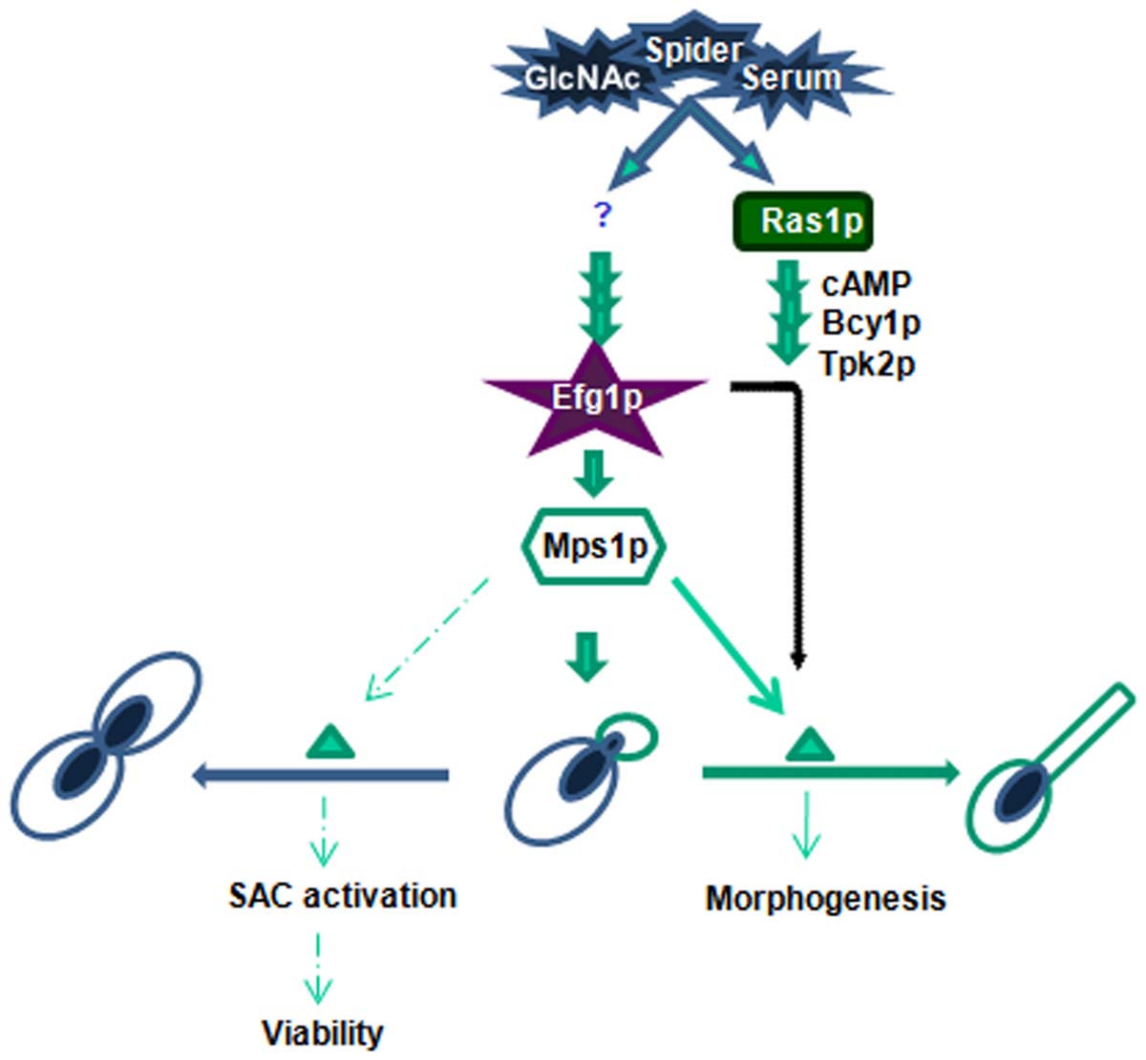
Model for illustration of possible regulatory mechanism for the activation of *MPS1* gene in *C. albicans*, in Efg1 dependent but Ras1 independent manner, which leads to morphogenesis as represented by lined arrows. A classical cAMP-PKA pathway is included for comparison. Role of Mps1 in SAC activation and ploidy maintenance is shown with dotted arrows. This model represents Efg1 as a conjunction point for SAC machinery and morphogenesis.

Further, in an attempt to unravel the functional link between morphogenesis and checkpoint signaling genes, we studied the *MPS1* expression in various filamentation inducing media and mutants of major signal transduction pathway involved in morphogenesis. qRT-PCR analysis revealed upregulation of *MPS1* in presence of Serum and N-acetyl Glucosamine in comparison to glucose grown cells. qRT-PCR analysis also revealed that *MPS1* expression is regulated by Efg1, in a Ras1, Tpk2 independent pathway. Further, it may be interpreted that the Efg1 dependent expression of a checkpoint kinase like *MPS1* suggests additional roles of Efg1 in regulating morphogenesis through spindle checkpoint signaling pathway (Figure 10). Morphological studies on various *in vitro* hyphae inducing conditions showed involvement of Mps1 in morphogenesis including chlamydospore formation in *C. albicans*. Mps1 knockouts also showed sensitivity towards oxidative stress. One of the mechanisms that *C. albicans* uses to evade macrophages is through morphological transition. Analysis of heterozygous *mps1* mutant (MFD2) engulfed by Macrophages showed presence of only yeast forms. Taken together, our experiments show that *MPS1* gene in *C. albicans* has obligatory functions in survival, checkpoint activation, morphogenesis and oxidative stress tolerance. The next step is to elucidate the interacting proteins which assist the functions of Mps1. It is also a future challenge to find whether nutrient signaling (like GlcNAc) and developmental processes in *C. albicans* are coordinated through spindle assembly checkpoint machinery, as shown in *Caenorhabditis elegans* [40].

## Supporting Information

Figure S1 **Computational characterization of Mps1p.** (**A**) Cartoon presentation for Kinase domain in the C-terminal side of the deduced amino acid sequence, was identified using motif finder at Expasy server. Computationally predicted Ser/thr kinase domain is located between aa 367 and 637 of Carboxy terminus. (**B**) Phylogenic tree constructed for studying evolutionary distances among the known and predicted *MPS1* family of protein kinases. The phylogram and bootstrap analysis were performed at Megatree server (http://www.megatree.com). Values at the start of branch point indicate calculated distances by peptide homology. Organisms and their respective gene name or accession numbers were provided. Sequences were downloaded from NCBI genome sequence depository.
(TIF)

Figure S2 **Mutant construction and essentiality confirmation of Mps1.** (**A**) A schematic presentation of construction of the cassette used for disrupting *MPS1*gene, by URA blaster technique. Disruption cassette, *mps1: hisG:: URA3::hisG:mp s1* was employed for inactivating the first allele, by homologous recombination was shown. Restriction enzymes used for preparing the cassette and DNA fragment used as probe for screening the transformants by southern analysis were also shown. (**B**) **A**utoradiogram for the confirmation of first allele mutants through *Pst*I enzyme digestion. The indicated 6.1 kb and 2 kb bands are after integration of *hUh* cassette in *MPS1*, and an undisrupted second allele respectively. Twelve positive transformants (MFD1-12) are shown in support of high efficiency of recombination. (**C**) Confirmation of essentiality by Heterozygote Trisome test. Disruption cassette, *mps1:Ura3':: ARG4::Ura3':mps1* used for inactivating first allele was schematically represented. An internal *cis*-recombination help to reconstruct active *URA3* gene from flanking Ura3' fragments. (**D**) Southern blot confirmation for first allele disruptants and triploids (*mps1*::*UAU1*/*mps1*::*URA3*/*MPS1*) obtained from HT test. In autoradiogram, a recurrent 2.3 kb band was observed in all the colonies screened; indicates triploids for the locus of *MPS1* gene which confirmed essentiality of the gene.
(TIF)

Methods S1 **Supplementary methods.**
(DOC)
