# Supplementary figures and images for: Characterization of a Putative Spindle Assembly Checkpoint Kinase Mps1, Suggests Its Involvement in Cell Division, Morphogenesis and Oxidative Stress Tolerance in Candida albicans
Source: PLoS One. 2014 Jul 15;9(7):e101517. doi: 10.1371/journal.pone.0101517 (PMC4098995; doi:10.1371/journal.pone.0101517)

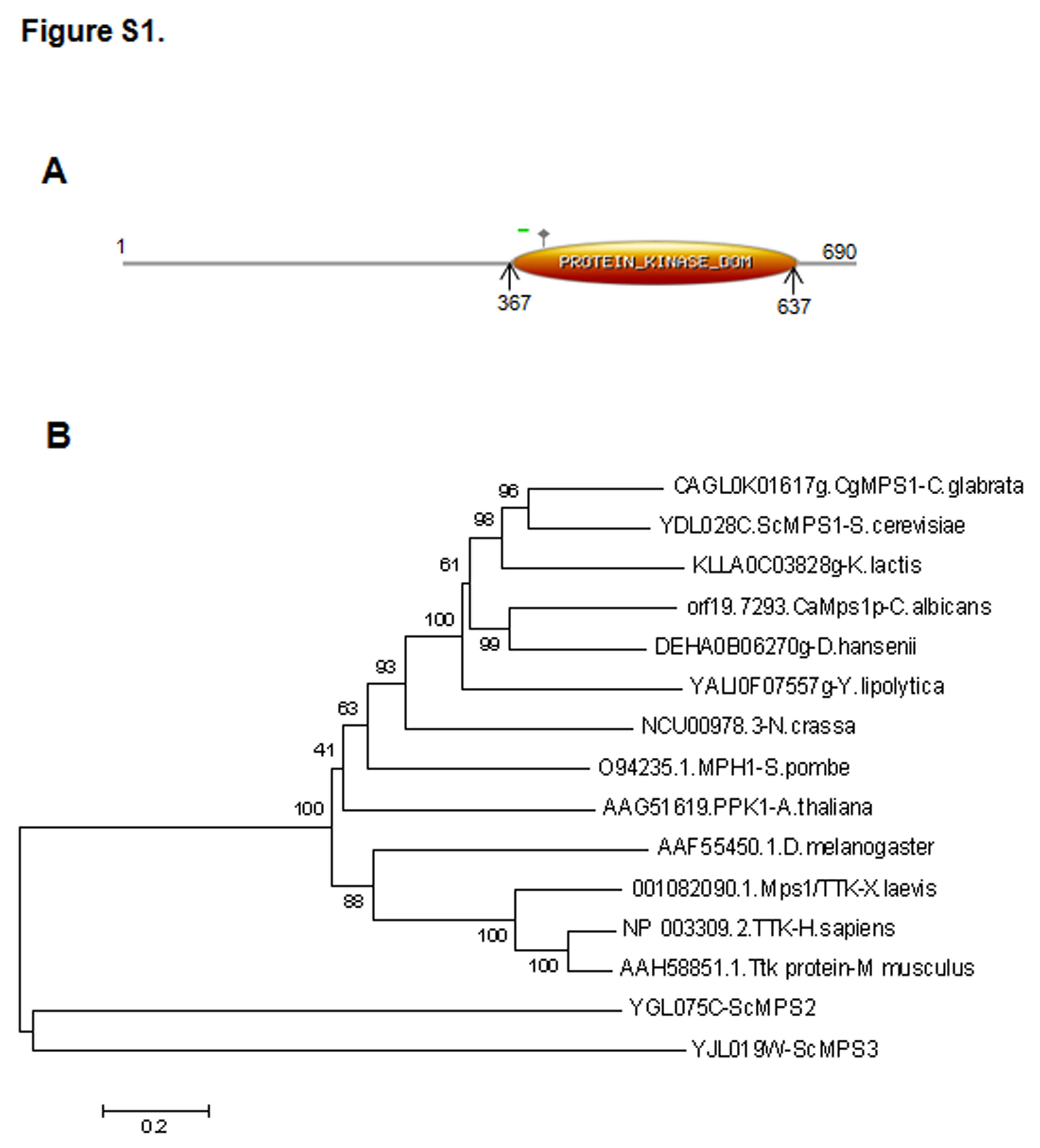

Supplement: Figure S1 — Computational characterization of Mps1p. (A) Cartoon presentation for Kinase domain in the C-terminal side of the deduced amino acid sequence, was identified using motif finder at Expasy server. Computationally predicted Ser/thr kinase domain is located between aa 367 and 637 of Carboxy terminus. (B) Phylogenic tree constructed for studying evolutionary distances among the known and predicted MPS1 family of protein kinases. The phylogram and bootstrap analysis were performed at Megatree server (http://www.megatree.com). Values at the start of branch point indicate calculated distances by peptide homology. Organisms and their respective gene name or accession numbers were provided. Sequences were downloaded from NCBI genome sequence depository. (TIF) [file pone.0101517.s001.tif]

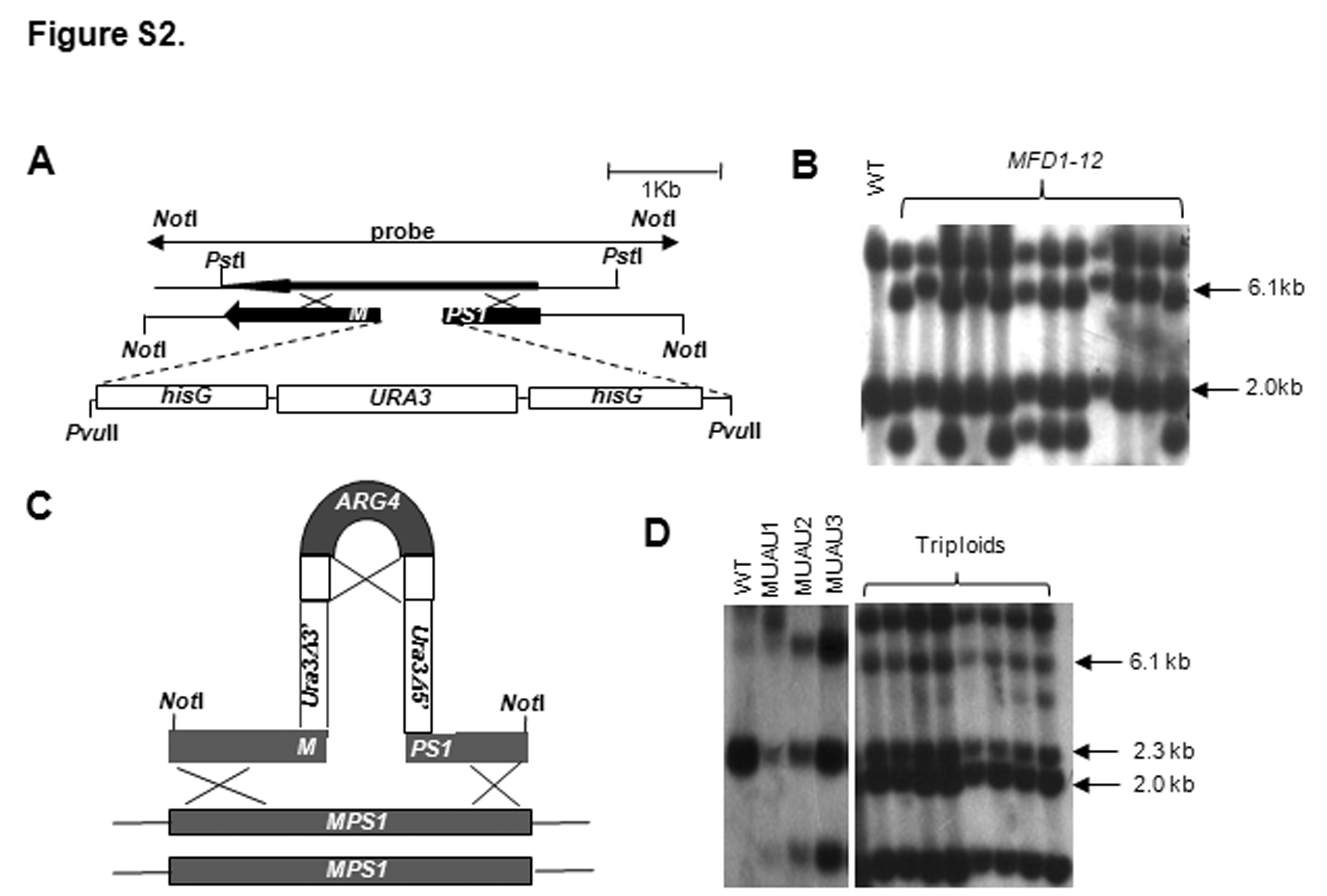

Supplement: Figure S2 — Mutant construction and essentiality confirmation of Mps1. (A) A schematic presentation of construction of the cassette used for disrupting MPS1gene, by URA blaster technique. Disruption cassette, mps1: hisG:: URA3::hisG:mp s1 was employed for inactivating the first allele, by homologous recombination was shown. Restriction enzymes used for preparing the cassette and DNA fragment used as probe for screening the transformants by southern analysis were also shown. (B) Autoradiogram for the confirmation of first allele mutants through PstI enzyme digestion. The indicated 6.1 kb and 2 kb bands are after integration of hUh cassette in MPS1, and an undisrupted second allele respectively. Twelve positive transformants (MFD1-12) are shown in support of high efficiency of recombination. (C) Confirmation of essentiality by Heterozygote Trisome test. Disruption cassette, mps1:Ura3':: ARG4::Ura3':mps1 used for inactivating first allele was schematically represented. An internal cis-recombination help to reconstruct active URA3 gene from flanking Ura3' fragments. (D) Southern blot confirmation for first allele disruptants and triploids (mps1::UAU1/mps1::URA3/MPS1) obtained from HT test. In autoradiogram, a recurrent 2.3 kb band was observed in all the colonies screened; indicates triploids for the locus of MPS1 gene which confirmed essentiality of the gene. (TIF) [file pone.0101517.s002.tif]
