# Supplementary material for: Characterization of a Putative Spindle Assembly Checkpoint Kinase Mps1, Suggests Its Involvement in Cell Division, Morphogenesis and Oxidative Stress Tolerance in Candida albicans
Source: PLoS One. 2014 Jul 15;9(7):e101517. doi: 10.1371/journal.pone.0101517 (PMC4098995; doi:10.1371/journal.pone.0101517)
Supplement: Methods S1 — Supplementary methods. (DOC) [file pone.0101517.s003.doc]

**Supplementary Information**

**Supplementary Methods**

To generate *mps1*∆ mutants URA blaster strategy was adopted, a 9.9Kbp plasmid pMP1 was constructed, by digesting the 4,322 bp fragment of *hisG-URA3-hisG* cassette from pUC19-CUB vector (Bisw*as et a*l., 2003b) and ligating with *Bst1107*I digested pGEM-MPS1 plasmid. Further, CAF3-1 strain was transformed by Lithium Acetate mediated transformation procedure (as described by Gie*tz et a*l., 1992), with a *Not*I digested, 6,915 bp fragment derived from the construct pMP1 (Figure S2A). Transformants were selected on SD-Uri agar media for Ura^+^ recombinants. First allele disruption was confirmed by Southern blot analysis using *Pst*I digestion (Figure S2B), one Ura^+^ transformant, MFD2 was used for Ura-curing process, on SD+Uri agar plates supplemented with 5’-fluoroorotic acid (5-FOA) (Sigma Aldrich) at 1mg/ml concentration. After confirmation by southern analysis, one Ura cured strain, MFD2-U1 was selected for further analysis.

A single transformation based gene function test (Enl*oe et a*l., 2000) was used to confirm the essentiality of *MPS1*. Plasmid pBME101 (a generous gift from Aaron P. Mitchell), was digested with *Pvu*II, to release a 4,217bp fragment containing, *Ura3’-ARG4-Ura3’* (for convenience, UAU) cassette. Further, this fragment was used to ligate with a 5,500bp *Bst1107*I digested plasmid, pGEM-MPS1 to obtain plasmid pMP2. Cassette from this final 6,717bp plasmid was digested by *Not*I digestion (Fig. S2C) and purified fragment was used for transforming BWP17 (*ura3∆/arg4∆/his1∆*) cells. Transformants were screened on SC+Uri-Arg plates at 30^o^C. Heterozygous mutants, MUAU1 to 30 (*MPS1*/*mps1*∆*::UAU*) were confirmed by Southern blot analysis. Further, for confirmation of essentiality, a single first allele mutant, MUAU1 was subjected to Homozygote Trisome test by growing. Mutant cells were grown to saturation in YPD lacking Uridine (YPD-Uri) for overnight at 30^o^C, for facilitating reconstruction of active *URA3* gene through *Cis*-recombinations between flanking *Ura3’* & *Ura5’* arms. A total of 1ml of each culture was washed twice with sterile water and a 100µl of such culture was used to plate on SC-Arg-Uri agar plates at 30^o^C for 2-3 days. A total of 75 colonies were screened by Southern blot analysis as described above (Fig. S2D).
